# Temporal Shifts in Cardiovascular Risk Factor Distribution

**DOI:** 10.1016/j.amepre.2013.10.011

**Published:** 2014-02

**Authors:** Gráinne H. Long, Rebecca K. Simmons, Margareta Norberg, Patrik Wennberg, Bernt Lindahl, Olov Rolandsson, Simon J. Griffin, Lars Weinehall

**Affiliations:** a MRC Epidemiology Unit, University of Cambridge, Cambridge, United Kingdom; b Department of Public Health and Clinical Medicine, Epidemiology and Global Health, Umeå University, Umeå, Sweden; c Department of Public Health and Clinical Medicine, Family Medicine, Umeå University, Umeå, Sweden; d Department of Public Health and Clinical Medicine, Occupational and Environmental Medicine, Umeå University, Umeå, Sweden; e Centre for Population Studies, Ageing and Living Conditions Programme, Umeå University, Umeå, Sweden

## Abstract

**Background:**

Complementary strategies to shift risk factor population distributions and target high-risk individuals are required to reduce the burden of type 2 diabetes and cardiovascular disease (CVD).

**Purpose:**

To examine secular changes in glucose and CVD risk factors over 20 years during an individual and population-based CVD prevention program in Västerbotten County, Sweden.

**Methods:**

Population-based health promotion intervention was conducted and annual invitation for individuals turning 40, 50, and 60 years to attend a health assessment, including an oral glucose tolerance test, biochemical measures, and a questionnaire. Data were collected between 1991 and 2010, analyzed in 2012 and available for 120,929 individuals. Linear regression modeling examined age-adjusted differences in CVD risk factor means over time. Data were direct-age-standardized to compare disease prevalence.

**Results:**

Between 1991–1995 and 2006–2010, mean age-adjusted cholesterol (men=−0.53, 95% CI=−0.55, −0.50 mmol/L; women=−0.48, 95% CI=−0.50, −0.45 mmol/L) and systolic blood pressure declined (men=−3.06, 95% CI=−3.43, −2.70 mm Hg; women=−5.27, 95% CI=−5.64, −4.90 mm Hg), with corresponding decreases in the age-standardized prevalence of hypertension and hyperlipidemia. Mean age-adjusted 2-hour plasma glucose (men=0.19, 95% CI=0.15, 0.23 mmol/L; women=0.08, 95% CI=0.04, 0.11 mmol/L) and BMI increased (men=1.12, 95% CI=1.04, 1.21; women=0.65, 95% CI=0.55, 0.75), with increases in the age-standardized prevalence of diabetes and obesity.

**Conclusions:**

These data demonstrate the potential of combined individual- and population-based approaches to CVD risk factor control and highlight the need for additional strategies addressing hyperglycemia and obesity.

## Introduction

There is a growing consensus that optimal preventive strategies to control type 2 diabetes and cardiovascular disease (CVD) should combine individual and population approaches—the former to identify and intervene in those at high risk of future disease, and the latter to instigate positive health changes in a large percentage of the population.^1^, ^2^, ^3^, ^4^ The International Diabetes Federation (IDF) endorsed this preventive approach for controlling modifiable risk factors and recommends targeting both people at high risk of diabetes and the entire population.^2^ However, a paucity of data support the beneficial effects of a combined individual and population approach for reducing population cardiovascular risk, with most data arising from theoretical and modeling studies.^2^, ^3^ This is largely due to a lack of longitudinal population risk distribution data and an overwhelming focus on individual high-risk approaches to prevention.^5^, ^6^ Because there is a positive continuous relationship between glycemia and CVD risk that extends below the diabetic threshold,^7^, ^8^, ^9^ the combined prevention approach holds great potential for reducing the macro-vascular complications associated with hyperglycemia in particular.

Data from the Västerbotten Intervention Programme (VIP)^10^ provide a unique opportunity to examine temporal changes in the distributions of glucose and other modifiable CVD risk factors in a Swedish middle-aged population when a combined individual and population prevention strategy was underway. Västerbotten County encompasses approximately 55,000 km^2^ of northern Sweden and had the highest CVD and mortality incidence in Sweden in the 1980s.^11^ VIP was introduced in 1985 to reduce the morbidity and mortality associated with diabetes and CVD.^10^ VIP combines population-based health promotion strategies, such as the introduction of a food labeling system denoting low-fat and high-fiber foods, with annual invitation for inhabitants turning 40, 50, or 60 years to attend a health assessment for systematic CVD risk factor screening and individual counseling by trained nurses. VIP was gradually introduced throughout Västerbotten and reached the entire middle-aged population by 1991.^10^ The aims of this study were to examine temporal changes in (1) the population distribution of plasma glucose, BMI, blood pressure, and cholesterol and (2) the prevalence of diabetes, dysglycemia, hypertension, hyperlipidemia, overweight, and obesity at a time when the individual- and population-based VIP prevention program was underway between 1991 and 2010.

## Methods

### Study Population and Data Source

Full details of the study population and VIP have been reported elsewhere.^10^ Briefly, annually since 1990, individuals turning 40, 50, or 60 years who live in Västerbotten County, Sweden, are invited for a health assessment at their local primary care center. Participation in the VIP increased from ~56% in 1995 to at least 66% since 2005. Minimal differences in age and education were observed between individuals who took part in VIP and those who did not. Initial small differences by sex and degree of urban residence decreased over time.^12^ Data were collected between 1991 and 2010, analyzed in 2012, and are available for 120,929 individuals. Numbers attending one, two, or three health assessments were 84,225, 35,629, and 1075, respectively. Written informed consent was obtained from VIP participants. Ethical approval was granted by the Regional Ethical Review Board, Umeå University.

### Individual Health Survey

The individual CVD risk factor screening and motivational interviewing by trained district nurses has been described previously.^10^ Briefly, VIP health assessments followed a detailed manual, used by all 36 health centers, that outlined procedures for extended examination, physician referrals, and/or counseling regarding lifestyle modification.^10^ Doctors were advised to follow current guidelines regarding pharmacologic intervention. A VIP educational seminar was attended annually by all primary care staff involved in the health assessments and included individual health promotion methods and any program updates. All primary care staff attended a motivational interviewing course, which uses open questions, active listening, and summaries to reinforce and encourage lifestyle changes. Evaluation work suggested that individual health surveys were important motivators of healthy lifestyle change.^13^

Participants attended their local health center after an overnight fast and completed a health questionnaire that included socioeconomic and demographic status, self-reported health, and lifestyle behaviors. BMI was calculated as weight (in kilograms) divided by the square of the height (in meters). Blood pressure (BP) was measured twice according to clinical guidelines with a mercury sphygmomanometer and the mean value used.^14^ Serum total cholesterol was analyzed from venous blood samples using Reflotron bench-top analyzers (Boehringer Mannheim GmbH).^15^ After an overnight fast, all participants not known to have diabetes and with fasting plasma glucose (FPG) <7.0 mmol/L were offered an oral glucose tolerance test (OGTT) performed according to the WHO criteria using a 75-g anhydrous glucose load.^16^ Glucose concentrations were measured on capillary plasma samples using Reflotron bench-top analyzers until 2004, after which Hemocue bench-top analyzers were used (Quest Diagnostics).

### Population-Based Prevention Program

The VIP population-based intervention aimed to raise public awareness of CVD risk factors and lifestyle behaviors.^10^ Interventions were tailored to the local community and integrated into everyday activities. They involved the whole community, from schools to retirement homes, and included the introduction of “the green keyhole” food-labeling system denoting low-fat and high-fiber foods, development of healthy school lunches, production and distribution of health educational materials, and health information meetings. Local activities, local distribution of health educational materials, and the food-labeling system were reported as influential motivators for healthy lifestyle change.^13^

### Measurements

Overweight was defined as 25≤BMI<30 and obesity as BMI≥30.^17^ Hypertension was defined as BP≥140/90 mm Hg^18^ or self-reported use of BP-lowering medication. Hypercholesterolemia was defined as cholesterol concentration≥5.2 mmol/L^19^ or self-reported use of prescribed lipid-lowering medication. As 2-hour plasma glucose (PG) measurements in capillary plasma yield higher values (on average 1.1 mmol/L) than in venous plasma, participants were assigned using the following WHO criteria^20^: impaired fasting glucose (IFG) was defined as FPG≥6.1 and <7.0 mmol/L and (if measured) 2-hour PG<8.9 mmol/L; impaired glucose tolerance (IGT) as FPG<7.0 mmol/L and 2-hour PG≥8.9 and <12.2 mmol/L; diabetes as FPG≥7.0 mmol/L and/or 2-hour PG≥12.2 mmol/L. The diabetes definition included self-reported diabetes and/or self-reported prescription of diabetes medication.

### Statistical Analysis

Results are expressed as mean±SD or number (%) where appropriate. Linear regression modeling examined, separately for each gender, age-adjusted differences in the means of continuous CVD risk factors over four calendar periods (1991–1995, 1996–2000, 2001–2005, and 2006–2010). Bonferroni post hoc comparisons assessed significant pairwise differences between calendar periods. To account for clustered data (repeat participation of some individuals), random-effects linear regression modeling with variance-corrected *β*-coefficients was used.

To examine glucose intolerance and related CVD risk factor prevalence, data were stratified by gender and age-standardized by the direct method using the Swedish 2011 population (Source: Statistics Sweden; www.scb.se/default____2154.aspx). Age-specific rates were calculated by summing the number of individuals with diabetes in each 5-year age group and dividing by the total population for that age group. Each age-specific rate was then multiplied by the standard population weighting for that age group and summed to produce the age-standardized rate. A two-sample test of proportions was used to assess differences in prevalence between calendar periods 1991–1995 and 2006–2010.

For all CVD risk factors, the correlation between population mean and corresponding risk factor prevalence was used to assess the extent to which population distributions shift as a whole (population mean and prevalence of high risk factor values are expected to correlate if the population distribution shifts as a whole^21^). The significance level was set at *p*<0.05. All analyses were performed using Stata (version 11.2) software.

## Results

### Population Characteristics

Table 1 presents the characteristics of 120,929 men and women VIP participants across four calendar periods 1991–1995, 1996–2000, 2001–2005, and 2006–2010. The mean age of participants was 49 years during 1991–1995 and 50 years during 2006–2010. The proportion of participants with middle or high education increased over time for both genders. Over the same calendar period and for both genders, the proportion of current smokers decreased, whereas the proportion of current snuff users increased. Self-reported use of glucose-lowering, lipid-lowering, and anti-hypertensive medication increased between 1991 and 2010 for both men and women (Table 1). Mean BMI exceeded 25 across all time periods and increased over time from 1991–1995 to 2006–2010 in both genders. Mean total cholesterol exceeded 5.20 mmol/L across all calendar periods but decreased over time from 1991–1995 to 2006–2010 in both men and women.

**Table 1:** Characteristics of 120,929 Västerbotten Intervention Programme participants by calendar period and gender

	**1991–1995**		**1996–2000**		**2001–2005**		**2006–2010**	
**Variable**	**Men (*n*=12,282)**	**Women (*n*=13,806)**	**Men (*n*=13,827)**	**Women (*n*=15,090)**	**Men (*n*=15,583)**	**Women (*n*=16,289)**	**Men (*n*=16,713)**	**Women (*n*=17,339)**
**Age (years)**	49.1 (7.9)	49.2 (8.0)	49.6 (7.8)	49.6 (7.9)	50.2 (8.1)	50.2 (8.0)	50.4 (8.2)	50.3 (8.2)
**Education**,^a^**%*****(n)***								
Low	36.8 (4,423)	36.4 (4,909)	29.7 (4,093)	27.7 (4,153)	21.3 (3,293)	19.4 (3,132)	15.4 (2,568)	13.0 (2,241)
Medium	44.7 (5,371)	40.3 (5,432)	49.7 (6,841)	44.5 (6,667)	55.3 (8,564)	47.4 (7,651)	57.6 (9,572)	47.5 (8,189)
High	18.4 (2,214)	23.3 (3,151)	20.5 (2,823)	27.8 (4,174)	23.3 (3,613)	33.2 (5,369)	27.0 (4,480)	39.5 (6,802)
**Marital status: married, %*****(n)***	82.1 (9,862)	81.9 (11,086)	81.6 (11,170)	82.1 (12,260)	79.7 (12,313)	80.7 (13,033)	78.7 (13,081)	80.3 (13,855)
**Smoking status, current, %*****(n)***	25.8 (3,091)	27.1 (3,663)	19.8 (2,681)	23.0 (3,429)	17.6 (2,682)	20.2 (3,251)	13.7 (2,242)	15.1 (2,580)
**Snuff user, current, %*****(n)***	21.1 (2,484)	1.6 (211)	25.4 (3,410)	3.9 (571)	28.8 (4,404)	7.7 (1,228)	26.9 (4,401)	9.4 (1,590)
**BMI**	25.9 (3.4)	25.3 (4.2)	26.3 (3.5)	25.6 (4.4)	26.7 (3.8)	26.0 (4.6)	27.1 (4.0)	26.0 (4.9)
**Fasting PG (mmol/L)**	5.4 (1.1)	5.3 (1.0)	5.6 (1.0)	5.5 (0.9)	5.8 (1.1)	5.6 (0.9)	5.6 (1.2)	5.5 (0.9)
**2-hour PG (mmol/L)**	6.3 (1.8)	6.9 (1.6)	6.4 (1.7)	6.9 (1.5)	6.6 (1.6)	7.1 (1.5)	6.6 (1.7)	7.0 (1.5)
**Total cholesterol** (**mmol/L)**	5.9 (1.2)	5.8 (1.2)	5.7 (1.1)	5.6 (1.1)	5.3 (1.0)	5.3 (1.0)	5.4 (1.0)	5.3 (1.0)
**Systolic BP** (**mm Hg)**	126.9 (18.7)	126.9 (18.7)	129.9 (17.0)	127.2 (18.8)	128.4 (16.9)	124.7 (18.3)	128.1 (15.9)	122.8 (16.7)
**Diastolic BP (mm Hg)**	81.6 (10.5)	78.6 (10.5)	81.2 (10.6)	78.0 (10.6)	79.2 (10.7)	75.6 (10.6)	80.5 (10.0)	76.3 (9.9)
**Prescribed medication, %*****(n)***								
Glucose-lowering drugs: yes	0.7 (85)	0.4 (51)	1.0 (134)	0.5 (81)	1.5 (234)	0.8 (134)	2.0 (332)	1.2 (197)
Lipid-lowering drugs: yes	1.3 (162)	0.6 (80)	2.7 (378)	1.1 (173)	5.2 (818)	3.0 (486)	7.8 (1,296)	4.5 (773)
Blood pressure– lowering drugs: yes	8.6 (1,056)	9.8 (1,354)	9.8 (1,356)	11.3 (1,705)	13.4 (2,091)	14.1 (2,305)	17.8 (2,970)	16.6 (2,872)

*Note:* Unless otherwise specified, data represent mean (SD).

a Completion of years in school: low=≤9; medium=10–12, and high≥13 years BP, blood pressure; PG, plasma glucose

### Temporal Changes in CVD Risk Factor Distributions

Mean age-adjusted total cholesterol fell by 0.53 mmol/L in men and 0.48 mmol/L in women between 1991–1995 and 2006–2010 (Table 2; *p*<0.001 for both comparisons). The direct age-standardized prevalence of hyperlipidemia decreased from 72.1% to 61.3% in men and from 66.9% to 55.1% in women over the same period (*p*<0.001 for both comparisons; Table 3). Similarly, there were declines in systolic and diastolic blood pressure (SBP and DBP; Table 2). Between 1991–1995 and 2006–2010 the mean age-adjusted decline in SBP was −3.06 mm Hg in men and −5.27 mm Hg in women (Table 2). Age-standardized prevalence of hypertension decreased from 38.3% to 34.3% in men and from 33.2% to 27.1% in women over the same time period (Table 3). Overall, temporal trends in CVD risk factors did not differ by SES (Appendix A, available online at www.ajpmonline.org), and adjusting for SES did not qualitatively change adjusted differences in continuous CVD risk factors over time (data not shown).

**Table 2:** Age-adjusted differences in continuous cardiovascular risk factors between 1991–1995 and (A) 1996–2000, (B) 2001–2005, and (C) 2006–2010

	**Risk factor β-coefficients (95% CI)**^a^			
**Variable**	**A**	**B**	**C**	***p*****-value**^b^
**Men**				
Fasting PG (mmol/L)	0.16 (0.13, 0.19)	0.32 (0.29, 0.34)	0.11 (0.80, 0.13)	A***, B***, C***, D***, E***, F***
2-hour PG (mmol/L)	0.07 (0.02, 0.11)	0.26 (0.22, 0.30)	0.19 (0.15, 0.23)	A*, B***, C***, D***, E***, F***
BMI	0.39 (0.31, 0.48)	0.78 (0.71, 0.85)	1.12 (1.04, 1.21)	A***, B***, C***, D***, E***, F***
Cholesterol (mmol/L)	−0.17 (−0.20, −0.15)	−0.61 (−0.63, −0.58)	−0.53 (−0.55, −0.50)	A***, B***, C***, D***, E***, F***
Systolic BP (mm Hg)	−0.60 (−0.99, −0.22)	−2.56 (−2.90, −2.22)	−3.06 (−3.43, −2.70)	A*, B***, C***, D***, E***, F*
Diastolic BP (mm Hg)	−0.55 (−0.80, −0.30)	−2.75 (−2.97, −2.53)	−1.46 (−1.70, −1.23)	A***, B***, C***, D***, E***, F***
**Women**				
Fasting PG (mmol/L)	0.13 (0.11, 0.15)	0.25 (0.23, 0.27)	0.12 (0.10, 0.14)	A***, B***, C***, D***, E^NS^, F***
2-hour PG (mmol/L)	0.06 (0.02, 0.10)	0.17 (0.14, 0.20)	0.08 (0.04, 0.11)	A**, B***, C***, D***, E^NS^, F***
BMI	0.29 (0.20, 0.39)	0.51 (0.43, 0.59)	0.65 (0.55, 0.75)	A***, B***, C***, D***, E***, F**
Cholesterol (mmol/L)	−0.16 (−0.18, −0.13)	−0.55 (−0.57, −0.53)	−0.48 (−0.50, −0.45)	A***, B***, C***, D***, E***, F***
Systolic BP (mm Hg)	−0.18 (−0.57, 0.21)	−3.27 (−3.60, −2.93)	−5.27 (−5.64, −4.90)	A^NS^, B***, C***, D***, E***, F***
Diastolic BP (mm Hg)	−0.75 (−0.99, −0.52)	−3.34 (−3.54, −3.14)	−2.71 (−2.92, −2.49)	A***, B***, C***, D***, E***, F***

*Note:* β-coefficients (95% CIs) are presented separately by gender.

a All regression models adjusted for age and clustering by VIP participant

b Bonferroni post hoc comparisons indicated significant pair-wise differences between calendar periods: A, 1991–1995 and 1996–2000; B, 1991–1995 and 2001–2005; C, 1991–1995 and 2006–2010; D, 1996–2000 and 2001–2005; E, 1996–2000 and 2006–2010; and F, 2001–2005 and 2006–2010, with significance levels indicated by *p<0.05, **p<0.001, ***p<0.0001 BP, blood pressure; PG, plasma glucose; VIP, Västerbotten Intervention Programme

**Table 3:** Direct age-standardized prevalence of glucose tolerance and cardiovascular risk factors of Västerbotten Intervention Programme participants

	**Calendar period**				
**Variable**^a^	**1991–1995**	**1996–2000**	**2001–2005**	**2006–2010**	***p*****-value**^b^
**Men (*n*)**	12,282	13,827	15,583	16,713	
Diabetes	4.7 (4.3, 5.1)	4.7 (4.4, 5.1)	6.4 (6.1, 6.8)	6.5 (6.2, 6.9)	<0.0001
IFG	6.7 (6.3, 7.2)	8.9 (8.4, 9.3)	14.8 (14.3, 15.4)	8.1 (7.8, 8.5)	<0.0001
IGT	3.9 (3.5, 4.2)	3.7 (3.3, 4.0)	5.0 (4.6, 5.3)	5.5 (5.1, 5.8)	<0.0001
Dysglycemia	15.2 (14.5, 15.8)	17.0 (16.4, 17.6)	25.9 (25.2, 26.6)	19.8 (19.2, 20.3)	<0.0001
Hypertension	38.3 (37.5, 39.1)	36.7 (35.9, 37.4)	33.4 (32.7, 34.1)	34.3 (33.6, 34.9)	<0.0001
Hyperlipidemia	72.1 (70.4, 72.0)	68.9 (68.2, 69.7)	55.9 (55.1, 56.7)	61.3 (60.5, 61.9)	<0.0001
Overweight	50.9 (50.1, 51.8)	56.0 (55.2, 56.8)	52.5 (51.7, 53.3)	52.7 (51.9, 53.5)	0.003
Obese	14.0 (13.5, 14.7)	19.8 (19.1, 20.5)	19.7 (19.1, 20.3)	22.7 (22.1, 23.4)	<0.0001
**Women (*****n*****)**	13,806	15,090	16,289	17,339	
Diabetes	3.6 (3.3, 3.9)	3.4 (3.1, 3.7)	4.2 (4.0, 4.6)	4.3 (4.0, 4.6)	0.0031
IFG	4.9 (4.6, 5.3)	6.0 (5.6, 6.4)	10.2 (9.7, 10.7)	7.0 (6.6, 7.4)	<0.0001
IGT	5.8 (5.4, 6.2)	5.7 (5.3, 6.1)	6.5 (6.1, 6.9)	6.5 (6.1, 6.8)	0.020
Dysglycemia	14.3 (13.7, 14.8)	15.0 (14.4, 15.6)	21.0 (20.2, 21.4)	17.5 (16.9, 18.0)	<0.0001
Hypertension	33.2 (32.5, 33.9)	33.0 (32.3, 33.7)	28.7 (28.1, 29.3)	27.1 (26.5, 27.7)	<0.0001
Hyperlipidemia	66.9 (66.1, 67.6)	63.8 (63.0, 64.5)	51.8 (51.1, 52.5)	55.5 (54.8, 56.2)	<0.0001
Overweight	36.2 (35.4, 37.0)	40.3 (39.5, 41.1)	36.3 (35.6, 37.0)	37.2 (36.5, 38.0)	0.056
Obese	16.8 (16.1, 17.4)	21.7 (21.0, 22.3)	19.4 (18.8, 20.0)	22.7 (22.0, 23.3)	<0.0001

*Note:* Age standardization was carried out using the direct method and the Swedish population on December 31st 2011 as the standard population (source: Statistics Sweden). Data are prevalence (95% CI).

a Full details on cut-offs used for categorizing glucose tolerance and CVD risk factors are presented in the Methods section

b Immediate form of the two-sample test of proportions was used to assess differences between calendar periods 1991–1995 and 2006–2010 IFG, impaired fasting glucose; IGT, impaired glucose tolerance

In general and regardless of the calendar period, men had higher FPG levels and women higher 2-hour PG levels. There were fluctuations between calendar periods in mean glucose concentrations, but on average both fasting and 2-hour PG values increased over time in both genders (Table 1, Table 2). Between 1991–1995 and 2006–2010, the age-adjusted mean increase in 2-hour PG was 0.19 mmol/L in men and 0.08 mmol/L in women. Over the same time period, the age-standardized prevalence of diabetes increased from 4.7% to 6.5% in men and from 3.6% to 4.3% in women (Table 3). Similarly, the age-standardized prevalence of IFG and IGT increased significantly in both genders over this time period (*p*<0.05). Mean age-adjusted BMI increased in both men (1.12) and women (0.65) between 1991–1995 and 2006–2010 (Table 2). The direct age-standardized prevalence of obesity increased from 14.0% to 22.7% in men and from 16.8% to 22.7% in women over the same period (*p*<0.001 for both comparisons) (Table 3).

**Figure 1:**
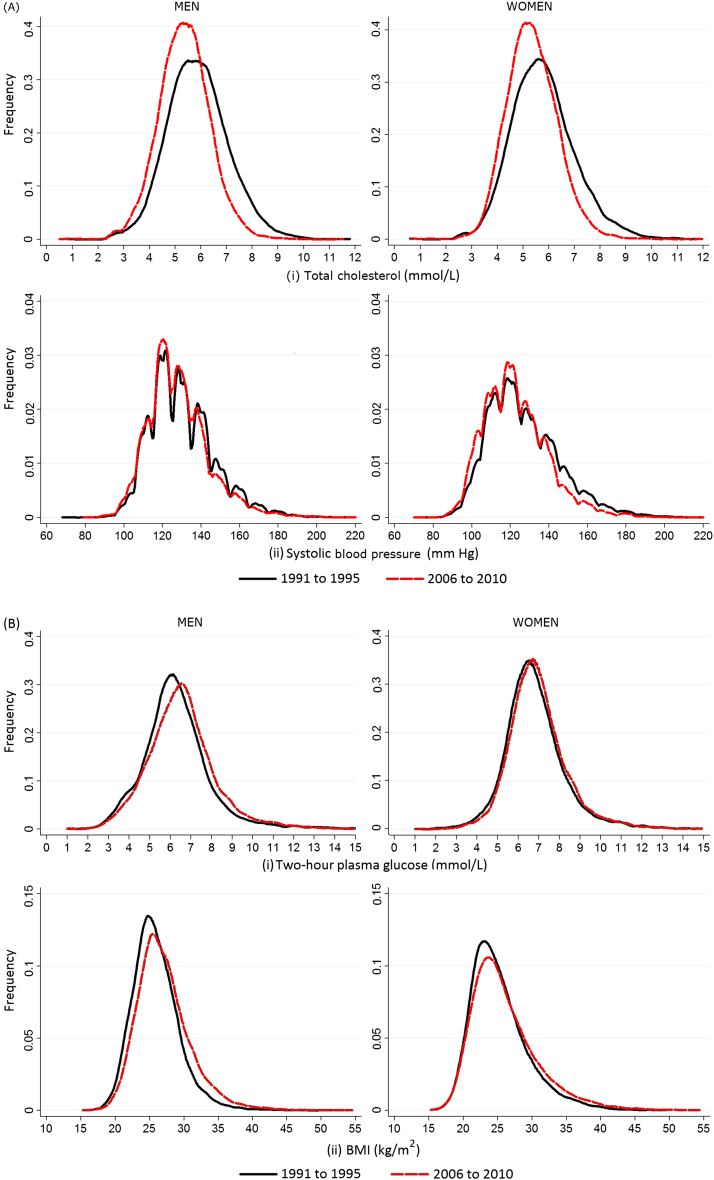
Distribution of cardiovascular risk factors in Västerbotten Intervention Programme participants between the calendar periods 1991–1995 and 2006–2010 in men and women. Plot (A) displays (i) cholesterol and (ii) systolic blood pressure and (B) displays (i) 2-hour plasma glucose and (ii) BMI

Figure 1 shows the distribution of risk factors in their continuous form in 1991–1995 and 2006–2010. Correlation between mean total cholesterol and prevalence of hyperlipidemia and mean SBP and the prevalence of hypertension was high (*r*=0.96 and *r*=0.95, respectively), supporting a downward shift in the entire population distribution of cholesterol and BP between these calendar periods. Similarly, high correlations between mean 2-hour PG and diabetes prevalence and mean BMI and obesity prevalence across calendar periods (*r*=0.94 and *r*=0.76, respectively) support an upward shift in the whole distribution of glucose concentrations and BMI.

## Discussion

Between 1991 and 2010, a downward shift in the entire population distribution of cholesterol and blood pressure was observed among the middle-aged population of Västerbotten County, with significant decreases in mean age-adjusted cholesterol and SBP. Over the same time period, an upward shift in the population distribution of glucose concentrations and BMI was observed, with significant increases in mean glucose and BMI levels. Alongside these population shifts, there were corresponding decreases in the age-standardized prevalence of hypertension and hyperlipidemia and increases in the prevalence of type 2 diabetes and obesity. The correlation between mean risk factor level and prevalence of the associated disease state across calendar periods was strong, supporting a shift in the age-standardized population distribution as a whole.^21^ It is likely that the combination of individual high-risk and population-based strategies employed by the VIP contributed to improvements in the population risk profile of cholesterol and BP. However, Northern Sweden faces a continued public health challenge in relation to the burden of disease associated with rising obesity and glucose concentrations.

Traditional preventive approaches often focus on targeting the high-risk minority. However, the majority of people exposed to low risk will experience more cardiovascular events than few people exposed to high risk.^4^ Consequently, there is a growing realization that complementary strategies, targeting high-risk individuals and the entire population, are needed to shift population risk factor distributions.^1^, ^2^, ^3^ Population-based strategies targeting the underlying determinants of disease are predicted to exert larger, cheaper, and more sustainable effects on population health and in so doing, reduce the number of high-risk individuals.^22^, ^23^ In Västerbotten County, glucose concentrations and BMI increased, not because the high-risk minority (right-hand tail of the population distribution) greatly increased their risk but because the whole population distribution moved upwards.^21^ A similar reverse argument applies to the downward shift in population levels of cholesterol and BP. Given that the association between hyperglycemia and CVD extends below the diabetes diagnostic threshold,^7^, ^8^, ^9^ continued individual and additional population strategies to curb increases in glucose and BMI are needed. Mass exposure controls, such as fiscal strategies for unhealthy foods or population health promotion programs, such as those employed in VIP, could change cultural norms and other underlying determinants of risk factor distributions and may exert additional effects on population health.

### Comparison with Other Studies

Declining levels of total cholesterol and BP were found in more than 66% of the 38 populations participating in WHO MONICA (MONItoring of trends and determinants in CArdiovascular disease) project from 1979 to 1996.^24^ This reflects wider Western secular trends in which declines in cholesterol, BP, and smoking have been linked to a substantial reduction in CVD mortality.^25^, ^26^ In the MONICA Northern Sweden population (Norbotten and Västerbotten counties), repeated population surveys revealed significant declines of 1.8 mm Hg for SBP and 0.8 mmol/L for total cholesterol in men, and 4.9 mm Hg and 0.9 mmol/L in women, respectively, between 1986 and 2009.^27^ Secular increases in the proportion of participants with middle- or high-education levels,^28^ reductions in the prevalence of smoking, and increases in leisure-time physical activity^29^ likely contribute to these secular improvements.

Although in the same direction of effect, improvements in BP and cholesterol in the MONICA Northern Sweden population are smaller than those in VIP, suggesting that VIP may have affected population levels of some CVD risk factors. It is likely that increased awareness, treatment, and management of CVD risk factors as a result of individual VIP assessments has contributed to improvements in risk factor levels and reduced the proportion with untreated high levels of CVD risk factors. In particular, increases in the use of BP-lowering^14^ and lipid-lowering^15^ medications and self-reported reduction in fat consumption up to 2004^30^ and increased physical activity levels^31^ in VIP participants may have contributed toward improved BP and cholesterol levels. However, the extent to which VIP contributed to improved CVD risk factor levels cannot be directly assessed here because of the lack of a suitable control group. An earlier quasi-experimental study comparing trends in risk factors between 1986 and 1995 in the Västerbotten municipality where VIP was initiated, to a comparable “control” population not participating in VIP, found that cholesterol and BP significantly improved in the intervention compared to the control population.^32^ This finding supports these results and underlines the challenge in addressing the rising prevalence of obesity and dysglycemia.

This finding of increasing levels of glucose intolerance confirm recent population studies in Sweden reporting adverse trends in blood glucose concentrations^33^, ^34^ and support the worldwide trend of increasing diabetes prevalence. Increases in diabetes prevalence may be due to an increase in diabetes incidence—although increases in incidence were not observed from 1972 to 2001 in central Sweden^35^—better treatment and/or detection of patients (e.g., as a result of screening in VIP); extended survival of diabetes patients; or as a consequence of an ageing population. It is also likely that rising obesity levels in Sweden have contributed to the rise in diabetes.^36^ Despite the rise in blood glucose concentrations, the frequency of diabetes in Sweden is lower than in most European countries.^37^ Although VIP may have attenuated the increases in glucose concentrations, it is not clear which specific aspects of the intervention program may have contributed to this.

An increase in mean BMI, as well as the prevalence of overweight and obesity, was observed in VIP. These results are consistent with previous Swedish studies,^38^, ^39^ including those in Northern Sweden.^40^, ^41^ Again, increases are comparatively lower than in other Western countries. Data from the WHO MONICA project suggest an increase of 0.5 in Northern Sweden between the early 1980s and mid-1990s compared to 1 and 1.5 in the United Kingdom and U.S., respectively.^41^ Increases in education^34^ and physical activity levels^31^ and decreases in saturated fat intake^29^ could help explain the slower rise in obesity in Västerbotten County. Again, the extent to which such changes occurred as a result of VIP could not be directly addressed in the present study.

Overall, previous VIP studies found that temporal trends in CVD risk factors did not substantially differ by SES, in agreement with these findings. Individuals from higher SES had lower BMI, glucose, cholesterol, and BP levels compared to lower social classes. Over calendar time, the gaps between socioeconomic groups reduced for BP and cholesterol but not for BMI and glucose.^14^, ^15^, ^31^, ^40^ Although minor differences in temporal trends of CVD risk factors between SES categories were found in the current study, overall temporal trends in risk factors were consistent across socioeconomic categories (Appendix A, available online at www.ajpmonline.org). The population-wide rise in Swedish education levels in recent decades^28^ may have minimized socioeconomic disparities in CVD risk factor levels and health over time. Differences between the findings here and previous VIP publications likely arise from differences in the time periods covered and in the method of analysis.

### Strengths and Limitations

A major strength of VIP is the large size (*n* >100,000) and long duration of follow-up (>20 years). This was achieved by integrating VIP into primary care, which helped facilitate data collection and long-term follow-up and ensured generalizability to similar locations. The use of OGTTs captured both clinically diagnosed prevalent undiagnosed diabetes cases, as well as changes in the population distribution of 2-hour glucose. A regular VIP training program for healthcare personnel and standard operating procedures ensured that measurements were comparable across centers and over calendar time. Limitations include the moderate participation rates and their large annual variation, which can introduce selection bias when nonparticipation is related to both exposure and outcome status. There may also have been error in the measurement of medication use, which was taken from a self-report questionnaire. Measurement protocols and laboratory analyses did vary over time, but appropriate adjustments were made following repeat testing of a sample of participants.^14^, ^15^

### Conclusion

The introduction of an individual- and population-based prevention program likely contributed to improvements in cholesterol and BP among middle-aged inhabitants of Västerbotten County, Sweden, between 1991 and 2010. Although the prevalence of diabetes and obesity has risen, estimates are lower than other comparative Western countries. These data demonstrate the potential for a combination of individual- and population-based prevention approaches to control CVD risk factor levels and highlight the need for additional strategies to minimize CVD risk and address the growing burden of disease associated with hyperglycemia and obesity.
