# Supplementary material for: Temporal Shifts in Cardiovascular Risk Factor Distribution
Source: Am J Prev Med. 2014 Feb;46(2):112–21. doi: 10.1016/j.amepre.2013.10.011 (PMC3898870; doi:10.1016/j.amepre.2013.10.011)
Supplement: Supplementary file 1 — Supplementary data [file mmc1.pdf]

Temporal Shifts in Cardiovascular Risk Factor Distribution

Gráinne H Long, PhD, Rebecca K Simmons, PhD, Margareta Norberg, MD, Patrik Wennberg, MD, Bernt Lindahl, MD, Olov Rolandsson, MD, Simon J Griffin, MD, Lars Weinehall, MD

Appendix A

Age–adjusted differences in continuous cardiovascular risk factors between 1991–1995 and (A) 1996–2000; (B) 2001–2005; and (C) 2006–2010, stratified by SES

		Risk factor β–coefficients (95% CI)[a]		
Variable (units)	SES	(A)	(B)	(C)
Men				
2-hour PG (mmol/l)	Basic	0.04 (−0.05, 0.12)	0.26 (0.18, 0.35)	0.11 (0.01, 0.21)
	Middle	0.08 (0.02, 0.14)	0.28 (0.23, 0.34)	0.21 (0.15, 0.27)
	High	0.08 (−0.01, 0.18)	0.25 (0.16, 0.33)	0.21 (0.12, 0.29)
BMI (kg/m^2)	Basic	0.29 (0.13, 0.45)	0.85 (0.70, 1.01)	1.19 (0.99, 1.40)
	Middle	0.48 (0.36, 0.61)	0.93 (0.83, 1.04)	1.37 (1.24, 1.49)
	High	0.48 (0.30, 0.66)	0.78 (0.63, 0.93)	1.12 (0.94, 1.29)
Cholesterol (mmol/l)	Basic	−0.22 (−0.27, −0.17)	−0.70 (−0.75, −0.65)	−0.62 (−0.68, −0.57)
	Middle	−0.17 (−0.21, −0.13)	−0.58 (−0.62, −0.55)	−0.48 (−0.52, −0.44)
	High	−0.05 (−0.11, 0.01)	−0.44 (−0.50, −0.39)	−0.41 (−0.46, −0.35)
Systolic BP (mm Hg)	Basic	−0.60 (−1.32, 0.13)	−2.01 (−2.74, −1.27)	−3.54 (−4.38, −2.70)
	Middle	−0.77 (−1.31, −0.22)	−2.24 (−2.72, −1.77)	−2.54 (−3.05, −2.02)
	High	0.71 (−0.12, 1.55)	−2.00 (−2.71, −1.30)	−1.52 (−2.28, −0.77)
Women				
2-hour PG (mmol/l)	Basic	0.15 (0.07, 0.23)	0.25 (0.17, 0.33)	0.19 (0.09, 0.29)
	Middle	0.02 (−0.03, 0.07)	0.18 (0.13, 0.23)	0.12 (0.07, 0.18)
	High	0.09 (0.03, 0.16)	0.20 (0.15, 0.26)	0.12 (0.06, 0.18)
BMI (kg/m^2)	Basic	0.34 (0.15, 0.52)	0.49 (0.31, 0.68)	0.88 (0.62, 1.13)
	Middle	0.49 (0.34, 0.64)	0.89 (0.77, 1.02)	1.19 (1.03, 1.34)
	High	0.35 (0.17, 0.53)	0.64 (0.50, 0.78)	0.74 (0.57, 0.91)
Cholesterol (mmol/l)	Basic	−0.23 (−0.28, −0.18)	−0.67 (−0.73, −0.63)	−0.68 (−0.73, −0.62)
	Middle	−0.13 (−0.17, −0.09)	−0.49 (−0.53, −0.46)	−0.41 (−0.44, −0.37)
	High	−0.03 (−0.08, 0.01)	−0.40 (−0.44, −0.36)	−0.30 (−0.34, −0.25)
Systolic BP (mm Hg)	Basic	−0.23 (−0.99, 0.52)	−3.44 (−4.19, −2.69)	−6.78 (−7.64, −5.90)
	Middle	0.35 (−0.23, 093)	−2.07 (−2.58, −1.56)	−3.85 (−4.41, −3.29)
	High	0.80 (0.10, 1.50)	−2.02 (−2.61, −1.43)	−2.95 (−3.60, −2.31)

β–coefficients (95% CI) are presented separately by gender. [a] All regression models adjusted for age and clustering by VIP participant.

VIP, Västerbotten Intervention Programme
